# Effects of acute warm-up intensity and baseline fitness on running performance in adolescents

**DOI:** 10.3389/fphys.2026.1819635

**Published:** 2026-06-18

**Authors:** Yongwan Kim, Hyun-chul Jeong, Suebin Park, Eun Young Song, Hyunji Kim, Juseok Yoon, Chansol Hurr

**Affiliations:** 1 Department of Physical Education, College of Education, Jeonbuk National University, Jeonju, Republic of Korea; 2 Center for Sports Science in Jeonbuk, Jeonbuk State Sports Council, Jeonju, Republic of Korea

**Keywords:** fitness level, heart rate, running performance, school-based intervention, warm-up intensity

## Abstract

This study investigated the interactive effects of acute warm-up intensity and baseline physical fitness on cardiovascular responses and subsequent running performance in adolescents within a school-based physical education setting. In a counterbalanced, repeated-measures crossover design, 132 adolescents completed a low-intensity warm-up (self-paced walking) and a moderate-intensity warm-up (modified interval-based circuit, ~70% estimated HRmax) on separate occasions, each followed by a 20-minute self-paced running task. After quality control, valid running performance data were obtained from 110 participants, and heart rate data were analyzed in a stratified subsample of 100 participants. Baseline fitness was categorized into middle-to-high fitness (grades 1–3) and low fitness (grades 4–5) based on national normative data from the Physical Activity Promotion System. Cardiovascular response, assessed as mean heart rate during the warm-up, and running performance outcomes, including total distance and mean speed, were analyzed using a mixed-effects model. A significant interaction between warm-up intensity and baseline fitness was observed for both total running distance (p = 0.027) and mean running speed (p = 0.030). Following the moderate-intensity warm-up, the middle-to-high fitness group demonstrated greater running distance and speed compared with the low-intensity condition, whereas the same standardized moderate-intensity warm-up did not improve performance in the low-fitness group. The moderate-intensity warm-up elicited higher mean heart rate than the low-intensity warm-up across participants (p < 0.0001), with no significant interaction between warm-up intensity and fitness level. These findings suggest that baseline fitness may moderate the performance response to an acute warm-up in adolescents. Overall, a universal warm-up prescription may not be equally effective across students with varying fitness levels, and individualized, relative-intensity approaches warrant further investigation in school-based physical education settings.

## Introduction

A warm-up serves as an acute physiological intervention that optimizes the transition from a resting to an active state (Bishop, 2003a; McGowan et al., 2015). Implementing a structured warm-up induces specific physiological adjustments, including elevated muscle and core temperatures, accelerated oxygen uptake (VO_2_) kinetics, and enhanced neuromuscular activation (Bishop, 2003b; Burnley and Jones, 2007). These metabolic and biomechanical responses improve muscle force production, increase tissue elasticity, and spare anaerobic capacity, thereby enhancing subsequent exercise performance (Fradkin et al., 2010; McGowan et al., 2015; Patti et al., 2022). The magnitude of these adaptations depends on the type, duration, and intensity of the warm-up, indicating that precise manipulation of warm-up parameters is required to optimize performance outcomes (Bishop, 2003b; Zois et al., 2011).

Warm-up intensity dictates the critical balance between physiological potentiation and fatigue accumulation. Evidence indicates that an optimal intensity threshold improves neuromuscular function and metabolic efficiency, translating to enhanced performance in both short-duration activities and middle-to-long-distance running (Alves et al., 2023; Ingham et al., 2013). However, protocols exceeding this threshold induce premature metabolic fatigue, such as glycogen depletion and excessive blood lactate accumulation, which compromise subsequent exercise capacity (González-Mohíno et al., 2018; Paris et al., 2021). These conflicting outcomes suggest that fixed or group-level warm-up prescriptions may not fully account for inter-individual differences in physiological responses. Instead, the physiological response to a given warm-up load may be moderated by individual characteristics, most notably baseline physical fitness (McGowan et al., 2015).

From a physiological perspective, baseline fitness may influence the relative demand imposed by a standardized warm-up. Individuals with higher aerobic capacity generally exhibit more efficient oxygen uptake kinetics and greater tolerance to exercise-induced fatigue (Burnley and Jones, 2007). Consequently, a moderate-intensity warm-up may provide an effective preparatory stimulus for a well-conditioned individual, whereas the same standardized workload may represent a relatively greater preparatory load for an individual with lower fitness and may therefore provide less favorable conditions for subsequent self-paced running (Bishop, 2003b; McGowan et al., 2015; Paris et al., 2021). Despite this theoretical rationale, empirical evidence detailing the interactive effects of warm-up intensity and baseline fitness remains largely limited to highly trained adult athletes under controlled laboratory or competitive conditions (Alves et al., 2023; Keesling et al., 2021).

Consequently, the applicability of previous findings to school-based physical education remains limited, as adolescents often exhibit heterogeneous fitness levels and distinct physiological maturation profiles (Faigenbaum et al., 2009; Malina et al., 2004; Jovanović et al., 2024). In typical educational environments, standardized warm-up routines are commonly applied uniformly, despite potential differences in individual fitness capacity. Despite extensive research on warm-up strategies, three issues remain insufficiently addressed. First, most studies examining warm-up intensity have focused on trained adults or athletic populations, whereas evidence in adolescents with heterogeneous fitness levels remains limited (Alves et al., 2023; Keesling et al., 2021). Second, previous studies have generally examined the main effect of warm-up intensity, but relatively little is known about whether baseline fitness moderates the performance response to the same warm-up protocol. Third, many prior studies have been conducted in laboratory or controlled athletic settings, which may not fully reflect real-world school-based physical education environments where warm-up routines are implemented at the group level (González-Mohíno et al., 2018; Alves et al., 2023; Jovanović et al., 2024). Therefore, it remains unclear whether a standardized moderate-intensity warm-up provides similar benefits across adolescents with different baseline fitness levels in an ecologically valid school-based setting.

Accordingly, the purpose of this study was to examine the interactive effects of acute warm-up intensity and baseline fitness level on cardiovascular responses and running performance in adolescents within a school-based setting. We hypothesized that a moderate-intensity warm-up would enhance running performance exclusively in adolescents with higher baseline fitness. Conversely, we postulated that adolescents with lower fitness would experience no performance benefits, as the same standardized warm-up may represent a less favorable preparatory stimulus for this group.

## Materials and methods

### Ethical approval and participants

A total of 132 adolescents from a public high school volunteered for this study. Following the experimental sessions, valid running performance data (total distance and mean speed) were successfully obtained for 110 participants. These participants were categorized into a middle-to-high fitness (MHF, n=65) group and a low fitness (LF, n=45) group based on their performance in a standardized national fitness assessment battery (detailed criteria and the assessment system are described in the ‘Baseline Fitness Assessment and Classification’ section below). While running performance was analyzed for all 110 participants, heart rate monitoring was restricted to a stratified subsample of 100 participants (20 per class, 4 per each fitness stratum) to ensure a balanced representation of physiological responses across the fitness spectrum. All participants and their legal guardians provided written informed consent, and the protocol was approved by the Institutional Review Board of Jeonbuk National University (No. 2025-02-017-002). Although pubertal status was not directly assessed, participants were recruited from a relatively narrow school-grade range corresponding to high school grades 1–2. This sampling strategy was intended to reduce, although not eliminate, between-participant variability in biological maturation.

### Study design and experimental procedure

This study employed a counterbalanced, repeated-measures crossover design to investigate the acute effects of warm-up intensity (low vs. moderate) on subsequent physiological responses and running performance. Prior to the main experimental sessions, participants underwent a one-to-two-week familiarization phase. During this period, students performed the identical 20-minute self-paced running task to minimize potential learning effects, establish reliable pacing strategies, and acclimate to the measurement procedures.

For the main experiment, participants were assigned to one of two counterbalanced sequences by class to control for potential order effects. Group A completed the moderate-intensity warm-up during the first session and the low-intensity warm-up during the second session, whereas Group B followed the reverse order. The two experimental sessions were separated by a one-week washout period to allow recovery and minimize potential carryover. All sessions were conducted during regular physical education classes, and external variables—including time of day, environmental conditions, instructional content, and the supervising instructor—were kept as consistent as feasible.

Each experimental session lasted approximately 50 minutes and followed a standardized sequence. The session commenced with a 10-minute warm-up protocol based on the assigned condition (detailed below). Heart rate was monitored continuously during the 10-minute warm-up phase and the subsequent 20-minute self-paced running task using the wrist-worn device. For the primary analysis of warm-up cardiovascular response, mean heart rate over the 10-minute warm-up phase was extracted. Heart rate during the subsequent 20-minute running task was also analyzed to characterize the overall cardiovascular response following each warm-up condition. Immediately following the warm-up, participants underwent a 5-minute transition period involving a slow-paced walk to relocate to the running track. This transition period was standardized across both warm-up conditions and was required to maintain the ecological structure of the school-based physical education class while ensuring that all participants began the running task under the same procedural conditions.

Following the transition, participants performed the main exercise: a 20-minute self-paced running task. The supervising instructor provided standardized verbal encouragement solely at the onset of the run, with no further external pacing guidance provided during the 20-minute period. Running performance was quantified using total running distance and mean running speed. The session concluded with a 5-minute standardized cool-down consisting of light walking and static stretching.

### Warm-up protocols

#### Low-intensity warm-up

The low-intensity warm-up served as an active control condition and consisted of 10 minutes of walking at a comfortable, self-selected pace. This protocol was designed to induce a gradual increase in baseline cardiovascular activation and promote local blood flow while minimizing substantial metabolic strain or neuromuscular fatigue.

#### Moderate-intensity warm-up

The moderate-intensity warm-up lasted 10 minutes in total, comprising a 2-minute dynamic mobility preparation phase followed by an 8-minute main interval circuit. This protocol was adapted from traditional interval training to safely achieve moderate cardiovascular arousal (targeting approximately 70% of estimated maximal heart rate) appropriate for a heterogeneous adolescent population. Although the protocol was designed to approximate a moderate relative intensity (~70% estimated HRmax), it was not individually calibrated using measured HRmax, heart rate reserve, ventilatory threshold, or perceived exertion. Therefore, in practice, the protocol functioned as a standardized group-level warm-up stimulus, allowing us to examine whether the same commonly applicable warm-up routine produced different responses according to baseline fitness level.

The 8-minute main circuit consisted of eight dynamic, multi-joint exercises: push-ups, planks, jumping jacks, bodyweight squats, side-steps, lunges, mountain climbers, and burpee jumps. Each exercise was performed for 20 seconds, followed by 10 seconds of passive rest. This circuit was structured to optimize whole-body neuromuscular activation and enhance subsequent biomechanical efficiency (Faigenbaum et al., 2006) while maintaining the 8-minute duration. The specific work-to-rest ratio and intensity were empirically established through pilot testing to ensure practical feasibility within the physical education curriculum. A detailed schematic of the exercise components is provided in **Table 1**.

**Table 1:** Detailed components of the moderate-intensity warm-up protocol.

Category	Exercise Program	Exercise Duration	Procedure	Intensity
Dynamic Mobility	Joint Mobility and Dynamic Stretching	2 min		
Moderate-Intensity Warm-up	Push-up	20 sec	4 min X 2 sets	70% of Maximum Heart Rate (HRmax 70%)
	Rest	10 sec		
	Plank	20 sec		
	Rest	10 sec		
	Jumping jack	20 sec		
	Rest	10 sec		
	Squat	20 sec		
	Rest	10 sec		
	Side-step	20 sec		
	Rest	10 sec		
	Lunge	20 sec		
	Rest	10 sec		
	Mountain climber	20 sec		
	Rest	10 sec		
	burpee jumps	20 sec		
	Rest	10 sec		

HRmax = estimated maximal heart rate. The main circuit follows an interval-based structure consisting of 8 dynamic multi-joint exercises. Each exercise is performed continuously for 20 seconds followed by 10 seconds of passive rest. The 8-exercise sequence (4 minutes) is completed twice for a total of 8 minutes.

### Measurements

#### Baseline fitness assessment and classification

Baseline physical fitness was evaluated using the Physical Activity Promotion System (PAPS), a standardized, nationally implemented fitness assessment battery for South Korean students (Lee et al., 2022). The PAPS evaluates multiple health-related fitness components, including cardiorespiratory endurance, muscular strength, flexibility, and body composition. Based on age- and sex-specific national normative data, students receive a composite fitness grade ranging from 1 to 5, with grade 1 indicating the highest fitness level and grade 5 indicating the lowest fitness level (Lee et al., 2017). Importantly, the PAPS grade represents a composite, norm-referenced fitness classification rather than a direct estimate of VO_2_max. Therefore, the grades cannot be directly converted into VO_2_max values.

In the present study, official PAPS evaluations were conducted at a certified National Fitness 100 center prior to the experiment. Based on these official records, participants were categorized into a middle-to-high fitness group (MHF; PAPS grades 1–3) and a low fitness group (LF; PAPS grades 4–5). This grouping was based on the standardized PAPS grading system and its practical use in the South Korean school context. According to national guidelines, students classified as grades 4–5 are identified as having low fitness and may be targeted for school-based health and fitness interventions (KICE, 2017). Thus, the classification was intended to distinguish students with nationally defined low fitness from those without low-fitness classification, rather than to represent a continuous physiological gradient. Although this classification is not directly interchangeable with VO_2_max-based categories used in other countries, it provides a nationally standardized and practically meaningful classification of adolescent physical fitness.

#### Heart rate

Cardiovascular responses were quantified using a wrist-worn photoplethysmography (PPG) device (Apple Watch Series 10, Apple Inc., Cupertino, CA, USA). To ensure a balanced representation of physiological responses across all fitness strata, a stratified sampling approach was utilized. Specifically, four students from each of the five PAPS grades within each class were assigned to wear the monitors, resulting in a monitored subsample of 100 participants. The devices recorded heart rate continuously during the 10-minute warm-up phase and the subsequent 20-minute self-paced running task. For the present analysis, mean heart rate during the warm-up phase was extracted to quantify the cardiovascular response to each warm-up condition. Mean heart rate during the subsequent running task was also analyzed to describe whether the cardiovascular response differed between warm-up conditions during the main exercise.

#### Running performance

Running performance was evaluated during the 20-minute self-paced running task using a GPS-based mobile application (Nike Run Club, Nike Inc., Beaverton, OR, USA) installed on each participant’s personal smartphone. This methodology was deliberately selected to preserve ecological validity and replicate authentic physical education conditions without the constraints of laboratory equipment. The application was activated at the onset of the run and terminated immediately upon completion. Total running distance (km) and mean running speed (km·h^−1^) were extracted as the primary performance outcomes. Because the task was self-paced, the total distance inherently accounted for any walking intervals utilized by the students. Trials exhibiting evident technical anomalies, such as GPS signal loss or incomplete recordings, were systematically excluded. Valid running performance data were successfully obtained for 110 participants.

### Validity and reliability

The Apple Watch used in the present study estimates heart rate using an optical photoplethysmography sensor and has demonstrated acceptable validity and reliability for monitoring heart rate during exercise (Khushhal et al., 2017; Nelson and Allen, 2019). Running performance was assessed using a GPS-based smartphone application (Nike Run Club, NRC). Previous studies have reported that GPS-based systems provide reasonably accurate estimates of distance under outdoor conditions, although accuracy may decrease at higher speeds, during rapid changes of direction, or in complex movement patterns (Jennings et al., 2010). Therefore, these devices were considered acceptable for field-based estimation of heart rate and running distance in the present ecological setting, while recognizing that they do not provide the same level of precision as laboratory-grade measurement systems.

### Statistical analysis

All statistical analyses were performed using GraphPad Prism software (GraphPad Software, San Diego, CA, USA). Descriptive data are presented as mean ± standard deviation (SD). Prior to inferential analyses, the assumption of normality for data distribution was verified using the Shapiro–Wilk test.

To evaluate the main and interactive effects of warm-up intensity and baseline fitness on physiological and performance outcomes, a two-way mixed-effects model utilizing restricted maximum likelihood was employed. Within this model, warm-up intensity (low-intensity vs. moderate-intensity) served as the within-subject repeated factor, while baseline fitness level (middle-to-high fitness vs. low fitness) was entered as the between-subject factor. The REML approach was specifically selected to robustly handle unbalanced datasets—resulting from missing valid recordings (heart rate, n = 100; running performance, n = 110)—without resorting to listwise deletion, operating under the assumption that data were missing at random.

Upon the detection of significant interaction effects, simple main effects analyses were subsequently conducted to delineate the differences between warm-up conditions within each specific fitness stratum. Where appropriate, *post hoc* pairwise comparisons were adjusted using the Bonferroni correction to strictly control for Type I error inflation. Effect sizes for main and interaction effects were calculated and are reported as partial eta squared (η²p). The alpha level for statistical significance was established *a priori* at p < 0.05.

## Results

### Cardiovascular responses

**Figure 1:**
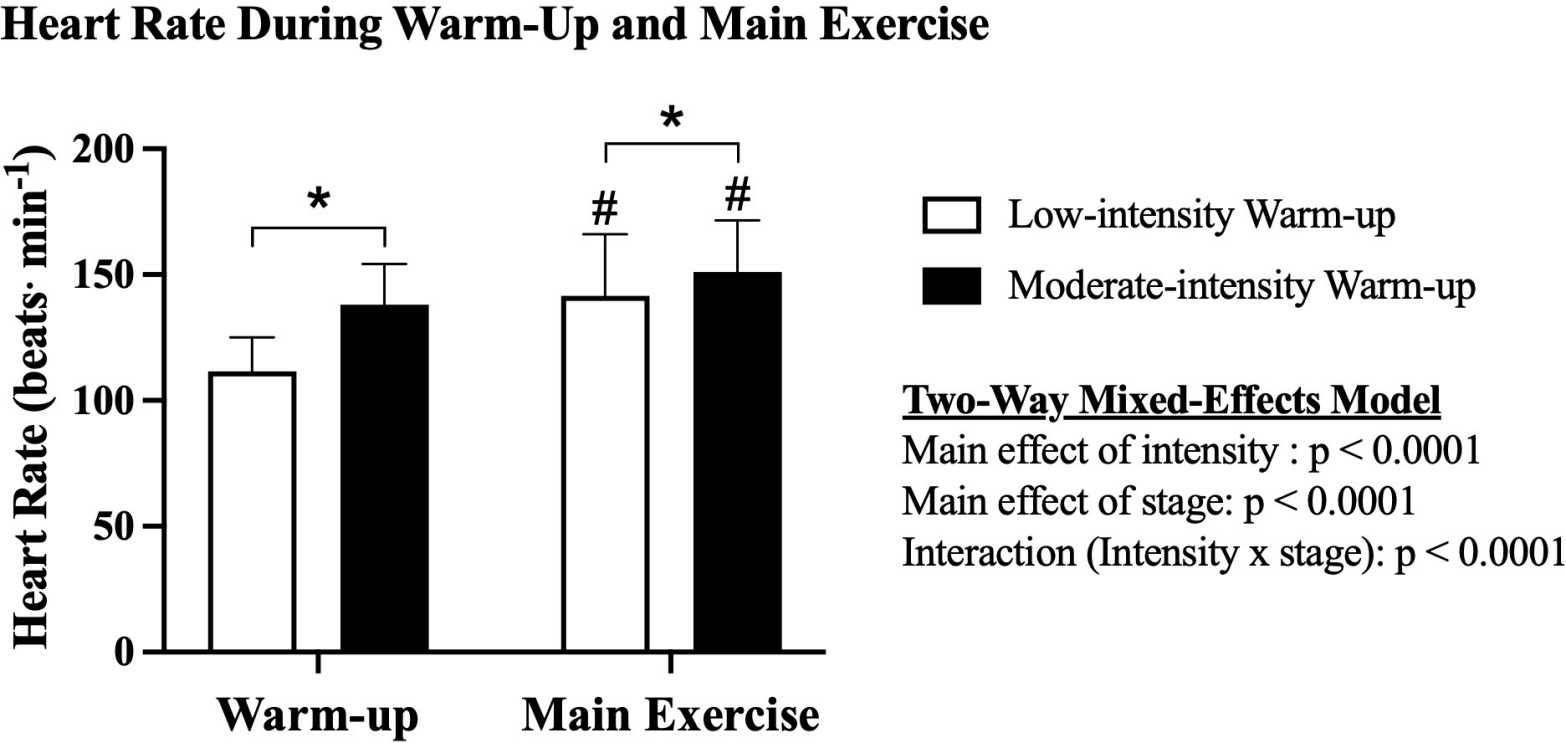
Cardiovascular responses during the warm-up and main exercise phases. Mean heart rate (bpm) was recorded during the 10-minute warm-up and the subsequent 20-minute self-paced main exercise following low-intensity and moderate-intensity warm-up conditions. Data were analyzed using a two-way mixed-effects model (REML). Significant main effects of warm-up intensity and exercise stage, as well as a significant interaction between intensity and stage, were observed (p < 0.0001 for all). Values are presented as mean ± SD (n = 100). * p < 0.05 between two warm-up intensity conditions; # p < 0.05 vs. warm-up HR within the same warm-up intensity condition.

To characterize cardiovascular responses during the warm-up protocols and subsequent main exercise, mean heart rates were analyzed (**Figure 1**). A two-way mixed-effects model revealed a significant main effect of warm-up intensity (p < 0.0001), a significant main effect of exercise stage (warm-up vs. main exercise, p < 0.0001), and a significant interaction between intensity and stage (p < 0.0001). During the warm-up phase, the moderate-intensity protocol elicited a significantly higher mean heart rate compared to the low-intensity protocol (138.1 ± 16.2 vs. 112.1 ± 13.5 bpm, p < 0.0001). This elevated cardiovascular activation was maintained during the main exercise, with the moderate-intensity condition resulting in a higher mean heart rate than the low-intensity condition (149.8 ± 20.5 vs. 142.7 ± 24.6 bpm, p = 0.0026).

**Figure 2:**
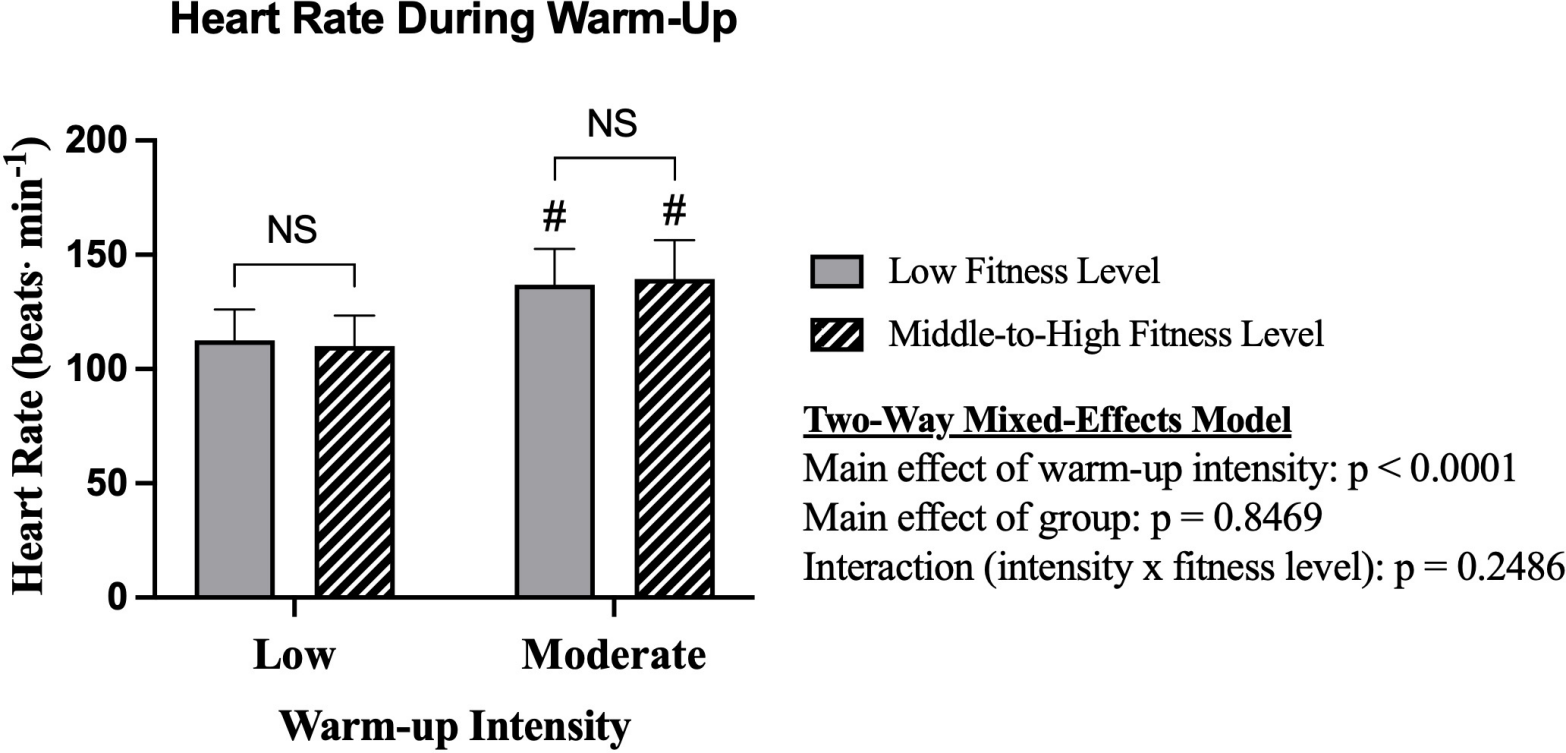
Mean heart rate during the warm-up phase stratified by baseline fitness level. Mean heart rate during the 10-minute warm-up is shown for the low-intensity and moderate-intensity conditions in the middle-to-high fitness (PAPS grades 1–3) and low fitness (PAPS grades 4–5) groups. Data were analyzed using a two-way mixed-effects model (REML). A significant main effect of warm-up intensity was observed (p < 0.0001), whereas no significant main effect of fitness group or interaction was detected. Values are presented as mean ± SD (MHF, n = 60; LF, n = 40). # p < 0.0001 vs. low-intensity warm-up within the same fitness group.

Furthermore, when analyzing the mean heart rate exclusively during the warm-up phase across fitness levels (**Figure 2**), a significant main effect of warm-up intensity was observed (p < 0.0001). However, there was no significant main effect of fitness level (p = 0.8469) nor a significant interaction between warm-up intensity and fitness level (p = 0.2486). Both the middle-to-high fitness group and the low fitness group exhibited comparable, significant increases in heart rate during the moderate-intensity warm-up compared to the low-intensity condition (p < 0.0001 for both). This indicates that the moderate-intensity protocol elicited similar absolute heart-rate responses across fitness groups.

### Running performance outcomes

#### Total running distance

**Figure 3:**
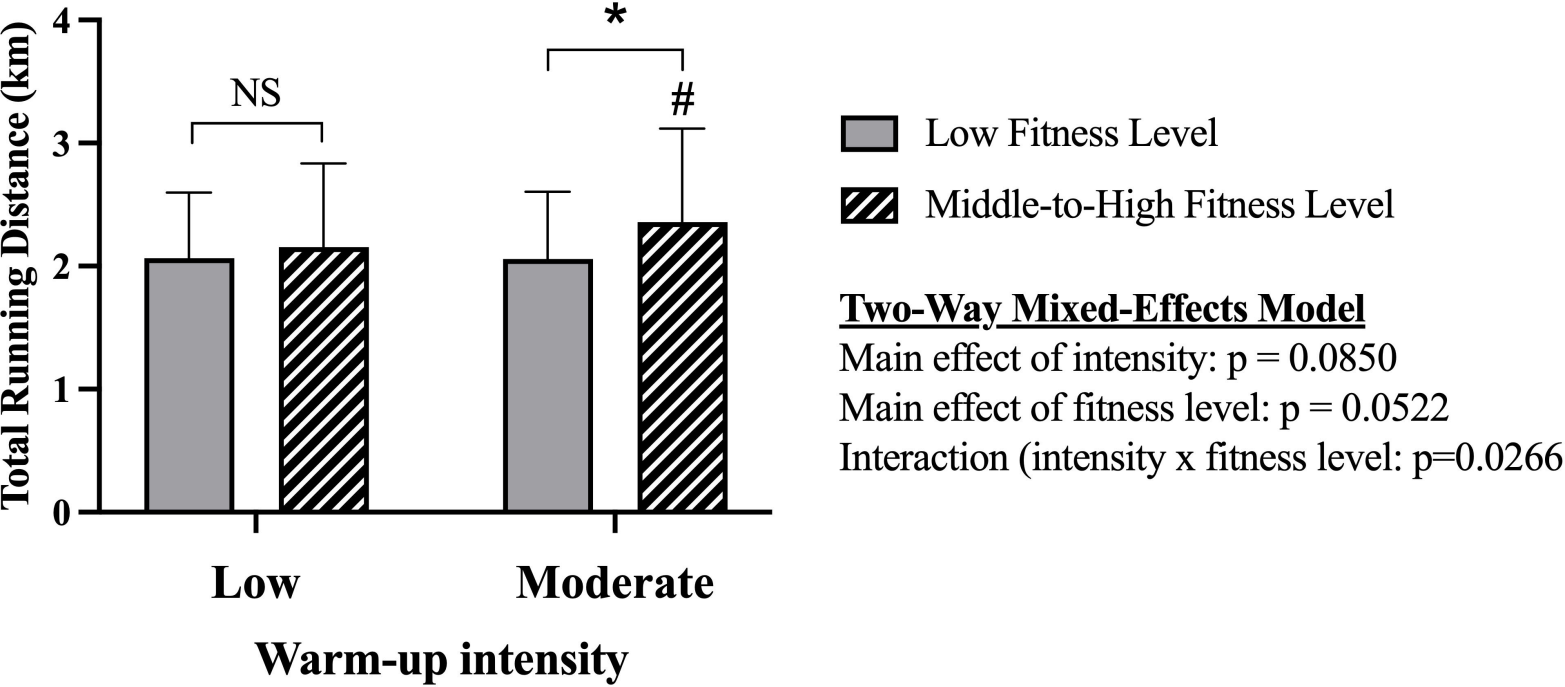
Interactive effects of warm-up intensity and baseline fitness on total running distance. Total running distance (km) completed during the 20-minute self-paced running task following low- and moderate-intensity warm-up conditions in the middle-to-high fitness (PAPS grades 1–3) and low fitness (PAPS grades 4–5) groups. Data were analyzed using a two-way mixed-effects model (REML). A significant interaction between warm-up intensity and fitness group was observed (p = 0.027). Values are presented as mean ± SD (MHF, n = 65; LF, n = 45). * p < 0.05 vs. low fitness level (Interaction (intensity × fitness level): p = 0.0266) within the same warm-up intensity condition; # p < 0.05 vs. low-intensity warm-up within the same fitness group.

A two-way mixed-effects model revealed a significant interaction between warm-up intensity and fitness level on total running distance (F(1, 96) = 5.07, p = 0.0266, η^2^p = 0.05; **Figure 3**). No significant main effects were found for warm-up intensity (p = 0.085) or fitness level (p = 0.052), indicating that the effect of warm-up condition differed according to baseline fitness level. The MHF group achieved a significantly greater running distance following the moderate-intensity warm-up compared to the low-intensity condition (2.34 ± 0.8 vs. 2.15 ± 0.7 km, p = 0.0091). Conversely, no significant difference was observed between warm-up conditions within the LF group (2.02 ± 0.5 vs. 2.05 ± 0.5 km, p = 0.6994). Additionally, following the moderate-intensity protocol, the MHF group covered significantly more distance than the LF group (p = 0.0075), whereas no such inter-group difference existed under the low-intensity condition.

#### Mean running speed

**Figure 4:**
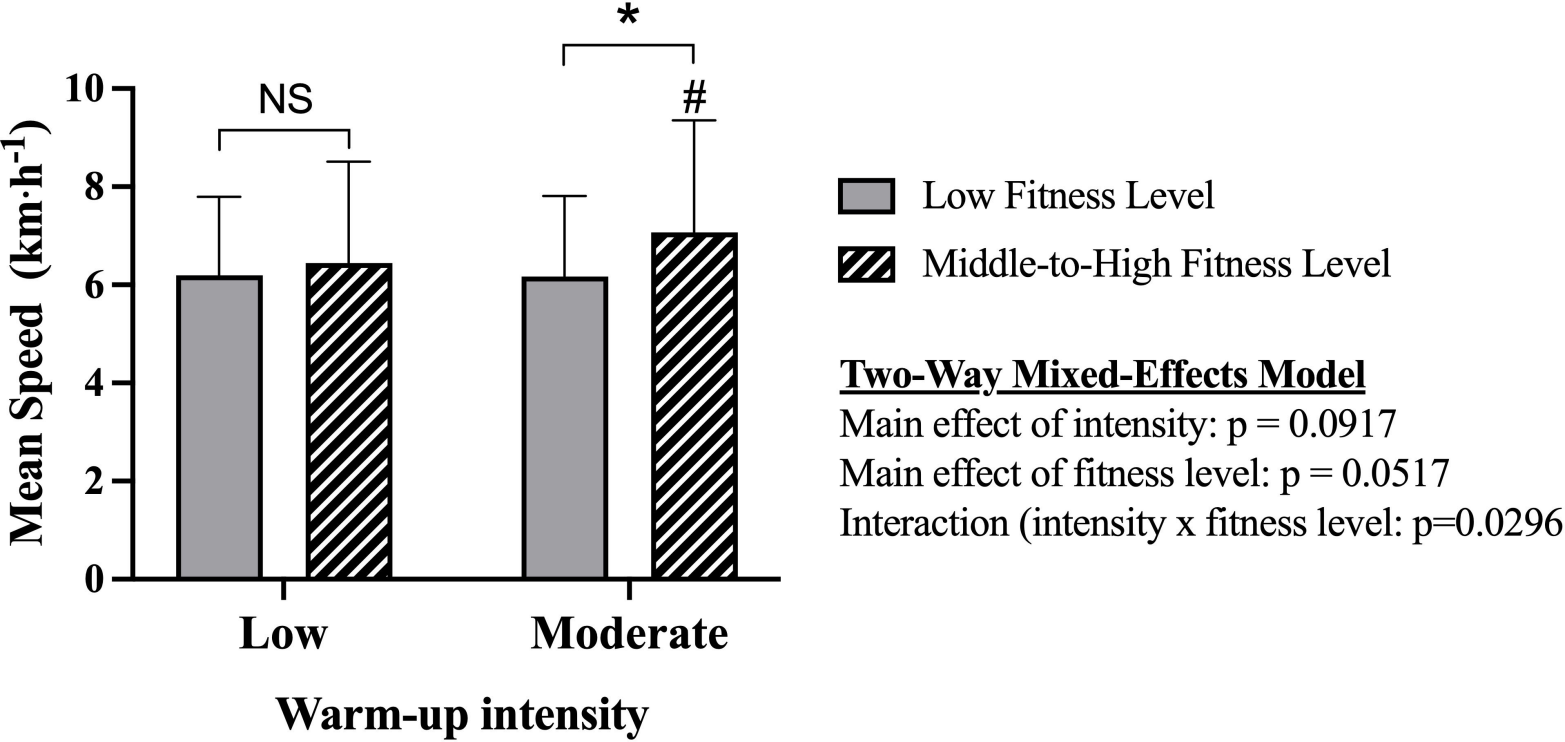
Interactive effects of warm-up intensity and baseline fitness on mean running speed. Mean running speed (km·h^−1^) achieved during the 20-minute self-paced running task following low- and moderate-intensity warm-up conditions in the middle-to-high fitness (PAPS grades 1–3) and low fitness (PAPS grades 4–5) groups. Data were analyzed using a two-way mixed-effects model (REML). A significant interaction between warm-up intensity and fitness group was observed (p = 0.030). Values are presented as mean ± SD (MHF, n = 65; LF, n = 45). * p < 0.05 vs. low fitness level (Interaction (intensity × fitness level): p = 0.0296) within the same warm-up intensity condition; # p < 0.05 vs. low-intensity warm-up within the same fitness group.

Similarly, a significant interaction between warm-up intensity and fitness level was observed for mean running speed (F(1, 95) = 4.87, p = 0.0296, η^2^p = 0.05; **Figure 4**). The main effects of warm-up intensity (p = 0.0917) and fitness level (p = 0.0517) were not statistically significant. *Post hoc* comparisons revealed that the middle-to-high fitness group achieved a significantly faster mean running speed after the moderate-intensity warm-up (7.01 ± 2.3 km·h^−1^) compared to the low-intensity warm-up (6.47 ± 2.1 km·h^−1^; p = 0.0109). In contrast, mean running speed in the low fitness group did not significantly differ between the moderate-intensity (6.07 ± 1.6 km·h^−1^) and low-intensity (6.14 ± 1.6 km·h^−1^) warm-up conditions (p = 0.7021). Following the moderate-intensity warm-up, the middle-to-high fitness group was significantly faster than the low fitness group (p = 0.0077).

## Discussion

The primary objective of the present study was to examine the interactive effects of acute warm-up intensity and baseline fitness on cardiovascular responses and running performance in a real-world, school-based physical education setting. Consistent with our hypothesis, the findings revealed a significant interaction between warm-up intensity and baseline fitness. A moderate-intensity warm-up significantly enhanced total running distance and mean running speed in adolescents with middle-to-high baseline fitness, whereas the same standardized warm-up protocol yielded no performance benefit in the low-fitness group. These divergent performance outcomes occurred despite both groups exhibiting comparable absolute heart-rate responses during the warm-up phase. The use of a standardized warm-up stimulus may help explain the observed interaction effect, as individuals with different baseline fitness levels responded differently to the same group-level workload.

Although the moderate-intensity protocol was intended to approximate 70% of estimated HRmax, it was not individually calibrated using measured HRmax, heart rate reserve, ventilatory threshold, or perceived exertion. Thus, the observed interaction should be interpreted as a differential response to the same practical, group-level warm-up protocol rather than as evidence that a precisely prescribed relative intensity produces different effects across fitness levels. This highlights the importance of considering inter-individual variability when interpreting warm-up effects. However, the observed interaction effect was modest in magnitude (η²p = 0.05) and should be interpreted with caution. Together, these findings suggest that the ergogenic efficacy of a warm-up may be moderated by individual fitness status, indicating that a standardized group-level warm-up prescription may not yield equivalent benefits across adolescents with heterogeneous fitness levels. The higher mean heart rate observed not only during the moderate-intensity warm-up but also during the subsequent running task suggests that the warm-up condition influenced the overall cardiovascular response across the session. However, because the interaction between warm-up intensity and fitness level was not significant for warm-up heart rate, the cardiovascular response alone does not explain the fitness-dependent differences in running performance.

The differential performance outcomes may be interpreted in light of the balance between warm-up-induced potentiation and fatigue (Bishop, 2003b; McGowan et al., 2015). However, this interpretation should be made cautiously because heart rate alone does not directly capture anaerobic contribution, peripheral muscle fatigue, blood lactate accumulation, or neuromuscular function (Buchheit, 2014; Burnley and Jones, 2007; Allen et al., 2008). For adolescents with middle-to-high fitness, the moderate-intensity warm-up may have provided an effective preparatory stimulus by increasing cardiovascular activation and facilitating readiness for subsequent running. However, because muscle temperature, VO_2_ kinetics, and neuromuscular responses were not measured, the precise mechanisms underlying the performance improvement cannot be determined from the present data.

In contrast, the absence of performance improvement in the LF group may indicate that the same standardized warm-up represented a less favorable preparatory stimulus for these participants. This finding is consistent with the concept of individualized warm-up, whereby the same warm-up protocol may impose different relative physiological demands depending on baseline fitness and the balance between potentiation and fatigue (Bishop, 2003b; McGowan et al., 2015). In low-fitness adolescents, a moderate-intensity circuit may have represented a relatively high exercise intensity, possibly approaching their ventilatory threshold (Burnley and Jones, 2007). If so, these participants may have begun the subsequent 20-minute running task with incomplete recovery from the warm-up, including residual cardiorespiratory or metabolic perturbations (Paris et al., 2021). However, because ventilatory threshold, blood lactate, VO_2_ kinetics, and neuromuscular fatigue were not directly measured, this interpretation should be regarded as a plausible explanation rather than a demonstrated mechanism.

The 5-minute transition period between the warm-up and running task should also be considered when interpreting the present findings. This interval was included for logistical reasons in the school-based setting and was standardized across conditions. Although such a delay may attenuate the immediate potentiation-related effects of a warm-up, previous evidence suggests that post-activation performance enhancement can persist across short recovery intervals, with recovery periods of approximately 3–7 minutes often allowing a balance between potentiation and fatigue dissipation (Wilson et al., 2013; Seitz and Haff, 2016). Therefore, the 5-minute transition may not have fully eliminated the preparatory effects of the moderate-intensity warm-up. However, because neuromuscular responses were not directly measured, we cannot determine whether the observed performance changes were specifically related to potentiation, fatigue dissipation, or other warm-up-related mechanisms. Notably, the MHF group still demonstrated improved running performance following the moderate-intensity warm-up, whereas the LF group did not. This pattern suggests that baseline fitness may have influenced the response to the same standardized warm-up protocol, although the physiological mechanisms underlying this difference cannot be determined from the present data.

From a practical standpoint, the present findings should be interpreted primarily within school-based physical education and adolescent sport settings. In these contexts, students or athletes often perform standardized warm-up routines despite having heterogeneous fitness levels. The present results suggest that teachers and coaches may consider adjusting warm-up intensity according to baseline fitness, using simple field-based indicators such as PAPS grade, shuttle-run performance, or other school-based fitness assessments. Adolescents with middle-to-high fitness may benefit from a short moderate-intensity dynamic warm-up, whereas low-fitness adolescents may require a shorter or lower-intensity warm-up guided by relative indicators such as heart rate, heart rate reserve, or ratings of perceived exertion. Any extension of these findings to clinical, rehabilitative, or pediatric disease populations should be considered speculative because the present study included healthy adolescents in a school-based setting.

## Limitations

We acknowledge several limitations. First, physiological responses were assessed primarily using heart rate during the warm-up phase. Although heart rate is a practical field-based indicator of cardiovascular strain, it does not directly capture anaerobic contribution, blood lactate accumulation, VO_2_ kinetics, ventilatory threshold, muscle temperature, or neuromuscular fatigue. Therefore, the mechanisms underlying the fitness-dependent performance responses cannot be determined from the present data. In addition, the moderate-intensity warm-up targeted approximately 70% of estimated HRmax but was not individually calibrated using measured HRmax, heart rate reserve, ventilatory threshold, or perceived exertion. Thus, the protocol should be interpreted as a standardized group-level warm-up stimulus rather than a precisely prescribed relative-intensity intervention.

Second, running performance was assessed using smartphone-based GPS measurements during regular physical education classes. This approach enhanced ecological validity but introduced greater measurement variability than laboratory-based or standardized GPS systems. GPS error, smartphone device variability, signal quality, group-based pacing behavior, motivation, and environmental factors may have influenced distance and speed estimates, potentially affecting effect sizes and interaction detection. Specifically, random measurement error associated with GPS signal variability may have reduced statistical sensitivity, whereas systematic differences in smartphone device models or group-based running patterns could potentially introduce directional bias in distance and speed estimates.

Third, pubertal status was not directly assessed and therefore could not be included as a covariate. Although participants were recruited from a relatively narrow school-grade range, residual maturational differences may have influenced fitness level, cardiovascular responses, and running performance. In addition, baseline fitness was dichotomized according to the PAPS grading system. While this policy-relevant classification was appropriate for distinguishing students with nationally defined low fitness, it may have reduced variability and obscured potential dose–response relationships across the five PAPS grades. Future studies should consider maturity status and treat fitness as a continuous or multi-level variable when sample size allows.

Finally, the present study examined only the acute effects of a single warm-up exposure. Therefore, it remains unclear whether repeated exposure to different warm-up intensities would produce similar responses or whether low-fitness adolescents might adapt to moderate-intensity warm-up protocols over time. Future studies using more controlled designs, direct physiological measurements, and longitudinal approaches are needed to confirm and extend the present findings.

## Conclusions

In conclusion, the present study suggests that baseline fitness may moderate the acute performance response to a standardized moderate-intensity warm-up in adolescents within a school-based physical education setting. A moderate-intensity warm-up improved running performance only in students with middle-to-high fitness, whereas the same standardized protocol did not improve performance in low-fitness students. This pattern may reflect differences in the relative physiological demand imposed by the warm-up; however, this explanation remains speculative because direct metabolic and neuromuscular markers were not measured. Given the modest interaction effect size, these findings should be interpreted as preliminary and require confirmation in future studies using more precise physiological and performance measurements.

## Data Availability

The raw data supporting the conclusions of this article will be made available by the authors, without undue reservation.
